# A Case of Endocarditis Secondary to Bacteremia Caused by Obstructive Pyelonephritis From Infective Nephrolithiasis in the Setting of Untreated Hyperparathyroidism

**DOI:** 10.7759/cureus.21788

**Published:** 2022-01-31

**Authors:** Zhongying An, Uzair Ashraf, Danilo Bacic Lima

**Affiliations:** 1 Internal Medicine, Rutgers University New Jersey Medical School, Newark, USA; 2 Cardiology, Rutgers University New Jersey Medical School, Newark, USA

**Keywords:** nephrolithiasis, bacteremia, hypercalcemia, infective endocarditis, primary hyperthyroidism

## Abstract

Hyperparathyroidism is known to be associated with nephrolithiasis but has less frequently been reported to contribute to infective endocarditis. We report a case of a 56-year-old woman with a past medical history of parathyroid adenoma, hyperparathyroidism, hypercalcemia, nephrolithiasis requiring bilateral nephrostomy, multiple episodes of complicated urinary tract infection (UTI), pulmonary and cardiac sarcoidosis treated with steroids previously, and intermittent complete heart block with implantable cardioverter-defibrillator (ICD), who presented to the ED with septic shock. She was found to have *Enterococcus faecalis* bacteremia complicated by large vegetations on her right ventricle ICD lead and tricuspid valve. Urinalysis was positive for leukocyte esterase, WBC, RBC, and bacteria. Transabdominal ultrasound and CT abdomen/pelvis showed multiple renal stones in bilateral kidneys. Nephrolithiasis secondary to untreated primary hyperparathyroidism had likely caused acute obstructive pyelonephritis in the patient, which had progressed to bacteremia and septic shock eventually leading to infective endocarditis. The patient was started on a six-week course of IV ceftriaxone and ampicillin, had her ICD removed, her blood cultures cleared, and was then referred to ENT and Endocrinology for parathyroidectomy. Prompt identification and treatment of hyperparathyroidism including parathyroidectomy can reduce the risk of nephrolithiasis and serious infections.

## Introduction

Hypercalcemia secondary to hyperparathyroidism is a common risk factor for nephrolithiasis. Large renal stones can cause ureteral obstruction leading to urinary stasis and urinary tract infection (UTI). Bacteria can ascend to the kidneys, resulting in pyelonephritis. If left untreated, bacteremia can travel through the bloodstream to distant sites in the body including the heart, and cause infection. The risk of endocarditis caused by hematogenous seeding increases with the presence of intracardiac devices such as implantable cardioverter-defibrillator (ICD). As indications and cardiac devices technology evolve, there will be an increased number of patients with ICDs. Approximately 80% of infective endocarditis cases are caused by *Streptococci *and *Staphylococci*. The third most common causative organism is *Enterococci*. We present an interesting case of a patient with infective endocarditis secondary to *Enterococcus faecalis *bacteremia caused by obstructive pyelonephritis from infected nephrolithiasis in the setting of untreated hyperparathyroidism.

## Case presentation

**Figure 1:**
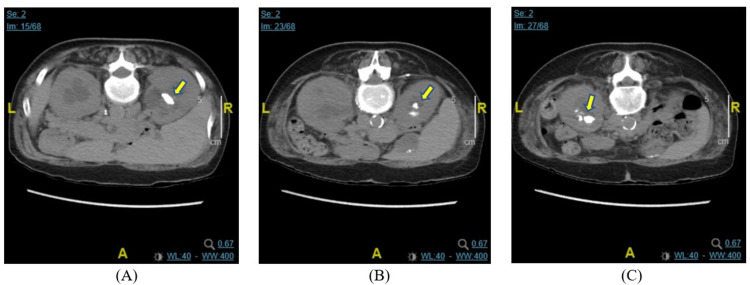
CT abdomen/pelvis without IV contrast in November 2020 Multiple stones within the right kidney with the largest ones measuring 1.8 cm in the upper pole (A) and an 8-mm stone within the lower pole (B). In the left kidney, there are multiple stones within the lower pole with the largest one measuring 1.5 cm (C) (arrows) CT: computed tomography; IV: intravenous

The patient was a 56-year-old woman with a past medical history of primary hyperparathyroidism secondary to parathyroid adenoma, hypercalcemia since 2016, bilateral nephrolithiasis complicated by recent *Enterococcus-*positive UTI requiring bilateral nephrostomy tubes, complicated history of coronary artery disease with prior non-ST segment elevation myocardial infarctions and placement of drug-eluting stents, sarcoidosis with pulmonary and cardiac involvement, as well as ICD placement two years prior to the admission due to paroxysmal complete heart block, and chronic obstructive pulmonary disease (COPD). She initially presented to the ED in November 2020 for a one-week history of bilateral flank pain and was found to have bilateral nephrolithiasis and *Enterococcus faecalis-*positive UTI requiring bilateral nephrostomy. Her corrected calcium was 11.9 mg/dL. CT abdomen/pelvis revealed bilateral large obstructing renal stones with largest stones measuring 1.8 cm in the right kidney and 1.5 cm in the lower pole of the left kidney with associated bilateral hydronephrosis (Figure 1). The patient underwent bilateral nephrostomy placement. Endocrinology recommended an outpatient Tc-99m sestamibi scan, but the patient was lost to follow-up after discharge. She then presented to the ED again in March 2021 with new-onset gross hematuria and was subsequently found to again have *Enterococcus-*positive UTI. She underwent a Tc-99m sestamibi scan, which suggested a parathyroid adenoma in the upper pole of the right thyroid. Subsequently, her nephrostomy tubes were removed. The patient had a history of elevated parathyroid hormone (PTH) at 159.8 pg/mL in 2018. Her parathyroid hormone-related protein (PTHrP), angiotensin-converting enzyme (ACE), thyroid-stimulating hormone, and free T4 were within normal ranges (PTHrP: <2.0 in 3/2021, ACE: <5 in 11/2020, TSH: 0.983 in 11/2020, and free T4: 1.0 in 11/2020).

**Figure 2:**
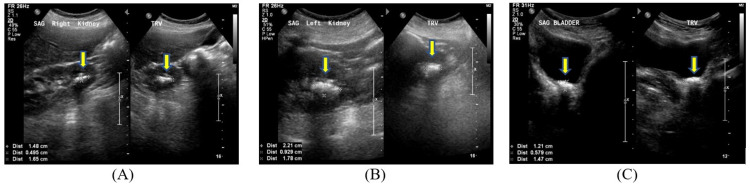
Transabdominal ultrasound of kidneys and urinary bladder Renal stones are present in the right kidney (A), left kidney (B), and left ureterovesical junction (C) (arrows)

**Figure 3:**
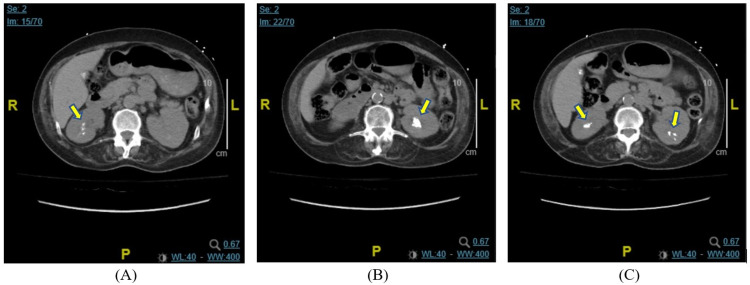
CT abdomen/pelvis without IV contrast in June 2021 Multiple renal stones are present in the bilateral lower poles of kidneys (arrows) CT: computed tomography; IV: intravenous

Two months after her discharge, the patient again presented to the ED after being found to have hypotension and hypoxia by a visiting nurse. She reported feeling chills, weakness, fatigue, and dizziness since April, which had become worse two days prior to the admission. She denied symptoms of flank pain, dysuria, hematuria, or urinary frequency. On presentation, the patient was afebrile, hypotensive at 83/61 mmHg, and tachycardic at 112 beats per minute. Physical exam was positive for bilateral costovertebral angle (CVA) tenderness. Labs were significant for brain natriuretic peptide (BNP) of 1,341 pg/mL, creatinine of 0.7 mg/dL (baseline: 0.3 mg/dL), troponin of 0.47 ng/mL, corrected calcium of 12.1 mg/dL, PTH of 371 pg/mL, erythrocyte sedimentation rate (ESR) of 82 mm/hr, C-reactive protein (CRP) of 192 mg/L, hypokalemia at K 2.8 mEq/L, and hypomagnesemia at 1.4 mg/dL. Blood culture grew *Enterococcus faecalis* (Table 1). Urinalysis showed moderate leukocyte esterase, WBC of 106/HPF, and RBC of 11/HPF (Table 1). Urine culture grew mixed flora with a colony count between 10,000 CFU/MI and 100,000 CFU/MI. Transabdominal ultrasound of kidneys and urinary bladder revealed multiple stones in the right kidney, left kidney, and left ureterovesical junction (Figure 2). CT abdomen/pelvis without IV contrast showed renal stones in the lower pole of bilateral kidneys and fullness of both renal collecting systems (Figure 3).

**Table 1:** Urinalysis

Variables	Results
Color	Yellow
Appearance	Slightly cloudy
Spec gravity	1.029
pH	6
Protein	30 mg/dL
Glucose	Negative
Ketones	Negative
Blood	Negative
Bilirubin	Negative
Urobilinogen	2.0 mg/dL
Nitrite	Negative
Leukocyte esterase	Moderate
RBC	11/HPF
WBC	106/HPF
Mucous threads	Rare
Bacteria	Rare
Squamepith	1/HPF

**Figure 4:**
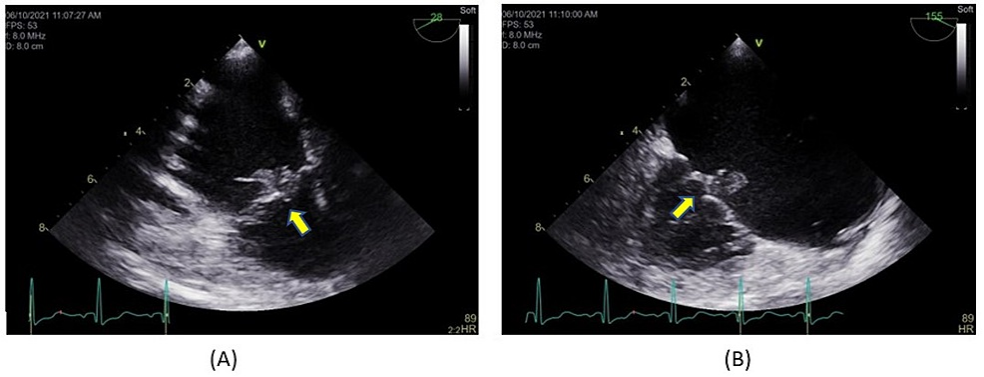
Parasternal long-axis view on transesophageal echocardiogram shows vegetation measuring 7.8 mm x 5.6 mm on the atrial aspect of the septal leaflet of the tricuspid valve (arrows)

**Figure 5:**
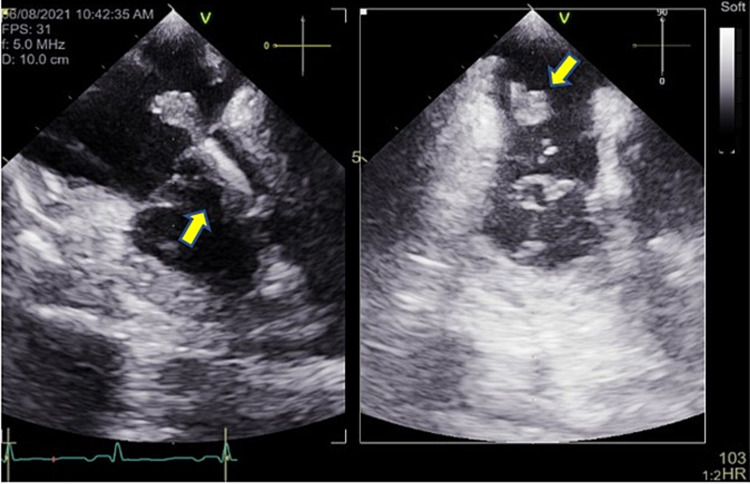
Parasternal long-axis view on transesophageal echocardiogram shows vegetation measuring 12 mm x 9 mm adherent to the atrial aspect of the ventricular lead of ICD in the right atrium (arrows) ICD: implantable cardioverter-defibrillator

One liter of normal saline was given with blood pressure improvement to 90/62 mmHg. The patient was initially started on broad-spectrum antibiotics, which were narrowed down to ampicillin/ceftriaxone based on bacteria susceptibility (Table 2). Transesophageal echocardiography revealed vegetation on the atrial aspect of the septal leaflet of the tricuspid valve measuring 7.8 mm x 5.6 mm (Figure 4) and vegetation measuring 12 mm x 9 mm adherent to the atrial aspect of the ventricular lead of ICD in the right atrium (Figure 5). The ICD was subsequently explanted. The patient was started on a six-week course of intravenous ampicillin/ceftriaxone. Her blood culture and urine culture were found to be negative after four days of being on antibiotics. She was given cinacalcet 30 mg for the treatment of hypercalcemia. Corrected calcium levels remained between 9 and 11 mg/dL. She was discharged with the advice to undergo outpatient follow-up with Endocrinology for the management of primary hyperparathyroidism, ENT for parathyroidectomy, Cardiology for surveillance, and follow-up for endocarditis and vegetations with repeat transthoracic echocardiography upon the completion of the antibiotic treatment.

**Table 2:** Susceptibility testing results for Enterococcus faecalis S: susceptible; R: resistant

Organism	Ampicillin	Erythromycin	Gentamicin 500	Levofloxacin	Rifampicin	Tetracycline
Enterococcus faecalis	S	R	R	R	S	R

## Discussion

Hypercalcemia secondary to primary hyperparathyroidism is often chronic. Patients with primary hyperparathyroidism and calcium level <12 mg/dL are often asymptomatic. The atypical presentation can range from symptomatic hypercalcemia to normocalcemia. Manifestations of hypercalcemia include bone pain, neuropsychiatric disturbances, constipation, nausea, nephrogenic diabetes insipidus, and nephrolithiasis. Symptoms can worsen as the calcium level increases. About 40% of patients tend to have hypercalciuria, a risk factor for renal stone formation. Nephrolithiasis, as in this case, occurs in approximately 15-20% of patients with primary hyperparathyroidism [1].

The initial diagnosis of primary hyperparathyroidism usually occurs with the incidental finding of elevated serum calcium levels. When total calcium concentration is elevated, a second- or third-generation PTH assay should be used to measure PTH levels to confirm the diagnosis of primary hyperparathyroidism [2]. About 80-90% of the patients with primary hyperparathyroidism tend to have elevated PTH levels. The rest have mildly elevated or normal PTH levels. The only definitive treatment for primary hyperparathyroidism is parathyroidectomy. Parathyroidectomy in patients with or without symptoms leads to the normalization of serum calcium concentrations [3]. Surgical treatment also lowers the risk of recurrent nephrolithiasis after surgery [4]. Parathyroidectomy should be recommended for patients with symptomatic primary hyperparathyroidism. Medical treatment with drugs inhibiting bone resorption can be considered in asymptomatic patients. Based on the American Association of Endocrine Surgeons Guidelines, parathyroidectomy is indicated, and is the preferred treatment, for all patients with symptomatic hyperparathyroidism [5].

This case is interesting as hyperparathyroidism is not a common risk factor associated with endocarditis. Our patient had a long history of elevated calcium and PTH levels since 2016, multiple episodes of complicated UTI, and infective nephrolithiasis requiring bilateral nephrostomy in 2020. However, her symptoms were not appropriately traced back to primary hyperparathyroidism. Nephrolithiasis secondary to primary hyperparathyroidism likely caused acute obstructive pyelonephritis in this patient, which progressed to *Enterococcus faecalis* bacteremia and septic shock eventually leading to infective endocarditis.

*Enterococcal *bacteremia rarely seeds distant organs or causes metastatic abscess [6]. However, it is often associated with endocarditis. *Enterococci*, most often *Enterococcus faecalis,* causes 5-15% of cases of infective endocarditis. Both normal and previously damaged heart valves can be infected, in which left-sided involvement is much more common than right-sided involvement [7]. *Enterococcal *infective endocarditis is usually subacute with nonspecific symptoms such as fever, chills, myalgia, weight loss, or decreased appetite. The diagnosis is based on clinical criteria (Tables 2, 3), a minimum of three positive sets of blood cultures, and echocardiography showing vegetation. Our patient was afebrile on presentation. However, she presented with septic shock and reported a few months' history of chills, weakness, and fatigue. Her blood cultures were positive for *Enterococcus faecalis*. Transesophageal echocardiogram showed vegetation on ICD leads and tricuspid valve. The prompt removal of the ICD device was the first step in infection-source control. Patients may or may need to be bridged with an external wearable defibrillator depending on the indication for ICD and risk of arrhythmia. This patient was not bridged, as her risk of arrhythmia was low. Multiple device interrogations at previous follow-up visits had shown no significant shockable arrhythmia or any prior history of delivery of tachy-therapy. Risk factors of infective endocarditis include preexisting valvular disease, congenital heart disease, history of prior infective endocarditis, intravenous drug use, indwelling intravenous catheters, immunosuppression, and recent dental procedures. Catheter-based aspiration of right-sided vegetations as a debulking procedure has been recorded in the literature, but no randomized controlled trials have yet been completed. Case reports have demonstrated that it can be a successful alternative when patients’ bacteremia does not clear.

**Table 3:** Duke criteria I IE: infective endocarditis

Description
Definite IE is established in the presence of any of the following:
Pathologic criteria
Pathologic lesions – vegetation or intracardiac abscess demonstrating active endocarditis on histology, OR
Microorganism – demonstrated by culture or histology of vegetation or intracardiac abscess
Clinical criteria
Using specific definitions listed in Table B:
2 major clinical criteria, OR
1 major and 3 minor clinical criteria, OR
5 minor clinical criteria
Possible IE
Presence of 1 major and 1 minor clinical criteria OR presence of 3 minor clinical criteria
Rejected IE
A firm alternative diagnosis is made, OR
Resolution of clinical manifestations occurs after ≤4 days of antibiotic therapy, OR
No pathologic evidence of IE is found at surgery or autopsy after antibiotic therapy for 4 days or less
Clinical criteria for possible or definite IE not met

**Table 4:** Duke criteria II IE: infective endocarditis; IgG: immunoglobulin G

Description
Major criteria
Positive blood cultures for IE (1 of the following):
Typical microorganisms consistent with IE from 2 separate blood cultures:
Staphylococcus aureus
Viridans streptococci
Streptococcus gallolyticus (formerly Streptococcus bovis), including nutritional variant strains (Granulicatella spp. and Abiotrophia defectiva)
HACEK group – Haemophilus aphrophilus (subsequently called Aggregatibacter aphrophilus and Aggregatibacter paraphrophilus), Actinobacillus actinomycetemcomitans (subsequently called Aggregatibacter actinomycetemcomitans), Cardiobacterium hominis, Eikenella corrodens, Kingella kingae
Community-acquired Enterococci in the absence of a primary focus, OR
Persistently positive blood culture:
For organisms that are typical causes of IE – at least 2 positive blood cultures from blood samples drawn >12 hours apart
For organisms that are more commonly skin contaminants – 3 or a majority of ≥4 separate blood cultures (with first and last drawn at least 1 hour apart)
Single positive blood culture for Coxiella burnetii or phase I IgG antibody titer >1:800
Evidence of endocardial involvement (1 of the following):
Echocardiogram positive for IE:
Vegetation (oscillating intracardiac mass on a valve or on supporting structures, in the path of regurgitant jets, or on implanted material, in the absence of an alternative anatomic explanation), OR
Abscess, OR
New partial dehiscence of prosthetic valve
New valvular regurgitation
An increase in or change in preexisting murmur is not sufficient
Minor criteria
Predisposition – intravenous drug use or presence of a predisposing heart condition (prosthetic heart valve or a valve lesion associated with significant regurgitation or turbulence of blood flow)
Fever – temperature ≥38.0 °C (100.4 °F)
Vascular phenomena – major arterial emboli, septic pulmonary infarcts, mycotic aneurysm, intracranial hemorrhage, conjunctival hemorrhages, or Janeway lesions
Immunologic phenomena – glomerulonephritis, Osler nodes, Roth spots, or rheumatoid factor
Microbiologic evidence – positive blood cultures that do not meet major criteria, OR serologic evidence of active infection with organism consistent with IE

## Conclusions

Although hyperparathyroidism is not a common risk factor for infective endocarditis, this case illustrates the importance of properly identifying and promptly treating hyperparathyroidism in preventing serious infections. Nephrolithiasis secondary to hyperparathyroidism can cause UTIs and obstructive pyelonephritis, which can progress to bacteremia. Bacteria can travel through the bloodstream and seed distant organs such as the heart leading to endocarditis especially in the setting of ICDs or other hardware. Asymptomatic hyperparathyroidism can be managed medically. It is imperative to recognize that when symptoms arise, a parathyroidectomy is indicated to prevent further morbidity and mortality.
